# Endocrine Disrupting Chemicals: Effects on Endocrine Glands

**DOI:** 10.3389/fendo.2019.00178

**Published:** 2019-03-21

**Authors:** Rosa Lauretta, Andrea Sansone, Massimiliano Sansone, Francesco Romanelli, Marialuisa Appetecchia

**Affiliations:** ^1^Endocrinology Unit, IRCCS Regina Elena National Cancer Institute, Rome, Italy; ^2^Section of Medical Pathophysiology, Food Science and Endocrinology, Department of Experimental Medicine, Sapienza Università di Roma, Rome, Italy

**Keywords:** endocrine disruptors, hormones, endocrine system, thyroid, ovary, testis, adrenal gland, pituitary gland

## Abstract

In recent years, endocrine disrupting chemicals have gained interest in human physiopathology and more and more studies aimed to explain how these chemicals compounds affect endocrine system. In human populations, the majority of the studies point toward an association between exposure to endocrine disrupting chemicals and the disorders affecting endocrine axis. A great number of endocrine disrupting chemicals seem to be able to interfere with the physiology of hypothalamus-pituitary-gonadal axis; however, every endocrine axis may be a target for each EDCs and their action is not limited to a single axis or organ. Several compounds may also have a negative impact on energy metabolic homeostasis altering adipose tissue and promoting obesity, metabolic syndrome, and diabetes. Different mechanism have been proposed to explain these associations but their complexity together with the degree of occupational or environmental exposure, the low standardization of the studies, and the presence of confounding factors have prevented to establish causal relationship between the endocrine disorders and exposure to specific toxicants so far. This manuscript aims to review the state of art of scientific literature regarding the effects of endocrine-disrupting chemicals (EDCs) on endocrine system.

## Background

In the last years, scientific literature has ever-increasingly focused on the role of endocrine-disrupting chemicals (EDCs) in human pathophysiology. A growing body of evidence, including reviews, clinical trials, and reports highlight new roles and effects of EDCs. EDCs are defined as “exogenous chemicals or mixture of chemicals that interfere with any aspect of hormone action” (1, 2). According to US Environmental Protection Agency (EPA), an EDC is an exogenous compound that may interfere with synthesis, secretion, transport, metabolism, receptor binding or elimination of endogenous hormones, altering the endocrine and homeostatic systems (3–8). In the process of defining EDCs criteria proposed by European Commission, it seems clear that EDCs should exhibit three actions: (1) endocrine activity; (2) deleterious and/or pathologic endocrine mediated-activity; (3) cause-effect relationship between substance and endocrine activity in exposed subjects (9, 10). Furthermore, European Food Safety Authority (EFSA) suggests that the most of EDCs are artificial substances that interfere with endocrine system binding hormonal receptors and/or regulating genomic expression (11). Indeed, epigenetic changes, such as DNA methylation and/or acetylation and histone modifications, appear to be involved in mechanisms related to endocrine disruption (2, 11, 12).

According to their origin, EDCs can be grouped as follows: industrial [i.e., dioxins, polychlorinated biphenyls (PCBs), and alkylphenols], agricultural (i.e., pesticides, insecticide, herbicides, phytoestrogens, fungicides), residential (phthalates, polybrominated biphenyls, bisphenol A), pharmaceutical (parabens) (5, 7, 13). Even heavy metals such as cadmium, lead, mercury, and arsenic may be included in the long list of EDCs (5, 14).

EDCs may display different routes to contaminate human body. Generally, inhalation, food intake and direct contact represent the most common exposure pathways (2, 7, 15). According to these pathways, EDCs may enter the food chain and accumulate in animal tissues up to humans (15). Most EDCs appear to be highly lipophilic, therefore accumulating in the adipose tissue—hence their generally long half-life (2, 16, 17). These two features explain the reasons why EDCs are able to accumulate for years in the adipose tissue of any animal, making co-contamination very frequent (18). Humans and other big mammals and top predators are at the top of food chain, therefore they may store larger doses of EDCs according to the process of bioaccumulation and bioamplification. These processes may generate a “cocktail” of effects with unknown consequences (19). Indeed, lifelong exposure to multiple compounds may bring about cumulative, additive and/or synergic effects. Dose-effect relationships can be complex for many EDCs causing diverging effects at different concentrations; moreover, different EDCs exert non-traditional responses to the dose due to the different action they can have in the binding of hormone receptors (1, 2, 4, 7, 18). Last but not least, the complexity of the hazardous effects of EDCs includes the concept of windows of vulnerability; indeed, evidence shows that the timing of exposure is of primary importance in the assessment of the effects on the endocrine system (1, 4, 7, 14, 18, 20).

Professional workers using pesticides, fungicides, and chemicals are at high risk of exposure to EDCs. Since 1996, the EPA has been constantly developing and promoting the Endocrine Disruption Screening Program (EDSP), which aims to: (1) develop protocols to research accurate, efficient and reproducible detection methods; (2) establish a priority regarding the substances to be studied; (3) assess practical guidelines in order to discriminate the correct detection test for any exposure condition (1, 2, 21).

## Endocrine Aspects of EDCs

The most documented mechanism in which EDCs act is represented by their ability to bind endocrine nuclear receptors (NRs) acting as total, partial, or inverted agonists or as antagonists (2–5). In particular, EDCs can bind to and activate various hormone receptors (androgen receptor, estrogen receptor, aryl hydrocarbon receptor, pregnane X receptor, constitutive androstane receptor, estrogen-related receptor, glucocorticoid receptor, thyroid hormone receptor, retinoid X receptor—AR, ER, AhR, PXR, CAR, ERR, GR, TR, RXR) and then mimic the natural hormone's action (3, 4, 13, 15). In some cases the same substance may activate certain receptor isoforms (i.e., agonist) and block other isoforms (i.e., antagonist), similarly to selective estrogen receptor modulators (SERM) behavior (14). Finally, EDCs may also interfere with the synthesis, transport, metabolism, and elimination of hormones, thereby decreasing the concentration of endogenous hormones (1, 3, 5). Furthermore, it should be considered that EDCs are able to affect endocrine system with addictive or synergistic effects as well. A specific EDCs may be innocuous *per se*, but its association with other EDCs may result in hazardous effects (i.e., cocktail effect) (6, 15, 18). The complexity of mixture toxicity, their interaction, and the presence of sensitive time windows make the risk assessment process extremely complex (7, 18, 22, 23). Every endocrine axis may be a target for each EDCs; their action is not limited to a single axis or organ but it is now clear that hypothalamus-pituitary gland-thyroid (HPT), hypothalamus-pituitary gland-gonads (HPG), hypothalamus-pituitary gland-adrenal (HPA) represent the main targets of EDCs (2, 7). Nevertheless, evidence shows that some EDCs may affect central nervous system (CNS) impairing hypothalamic and pituitary functions, therefore disrupting the proper actions of peripheral glands with unclear effects (2, 5, 7). Recent evidence shows that EDCs may disrupt energy metabolic homeostasis altering adipose tissue (increase in number and size of adipose cells), impairing endocrine regulation of adipose tissue and adipocytokine production, reducing basal metabolic rate and changing the regulation of appetite and satiety (24). This happens especially if the exposure occurs during early development. These chemicals, called “obesogens,” could predispose some people to weight gain despite following a low caloric diet and increasing physical activity (4, 16, 25). For example, Diethylstilbestrol (DES) is an estrogenic chemical binding with high affinity to both ERα and ERβ which plays an important role in adiposity regulation as well as central and peripheral energy balance. Developmental exposure to DES in mice induces adipogenesis and causes mice to become obese or overweight (2, 16). Indeed, the onset of many chronic metabolic diseases, such as obesity, diabetes, hypertension, and dyslipidemia may be accelerated by chronic, low-dose exposures that characterize human contact with environmental toxicants and their interactions (2, 16, 17). Recent data point out that exposure to EDCs during development can not only cause direct damage to the exposed individual, but also to the individual's offspring and to future generations—a process that is called transgenerational inheritance. From these evidences a new paradigm for non-communicable disease has been postulated: the evolutionary origins of health and disease (DOHaD).

Recent evidence has shown in several mammals, including humans, how EDCs can influence behavior and how this interaction is different in both sexes. There were differences in males and females after exposure to BPA *in utero* and early postnatal life. Recent literature has shown that chromosomal sex is a fundamental variable in accounting for the effects of BPA on behavior. Several studies have confirmed the ability of BPA and other EDCs to influence rodent brain development in a gender-specific way, even at very low doses, altering the normal steroidegenesis programmed in the two sexes through epigenetic alterations that can lead to differential gene expression (26). Even in human epidemiological studies, it appears that BPA has specific effects on both sexes. Prenatal BPA levels are positively associated with an increase in externalizing behaviors in girls, against greater internalizing behavior, anxiety, and aggression in boys. Because several neuropsychiatric disorders show a gender-specific incidence, it is important to understand how hormones and other substances modify neurobehavioral dimorphisms.

## Pituitary Gland

The diencephalic system represents a preferential target of EDCs, which may alter proper function of CNS mimicking neurotransmitter actions, beside their ability to bind endocrine receptors (27). Several EDCs act on the pituitary gland, therefore influencing the different endocrine axes: as a result, a wide spectrum of clinical manifestations has been associated with exposure to pollutants, such as precocious/delayed puberty (28) and circadian disruption (29).

## Reproductive System

As the chemical structure of most of EDCs mimics sex gonadal hormones and has the ability to bind to endocrine receptors interfering with hormonal signals, reproductive system represents the most vulnerable endocrine axis to EDCs actions (13). The US EPA described five classes of EDCs endowed with anti-androgenic properties and, simultaneously, with weak estrogenic activity: (1) drugs or synthetic estrogens (i.e., 17 β estradiol, diethylstilbestrol), (2) phytoestrogens (i.e., isoflavonoids, cumestans, lignans, stilbens), (3) pesticides (i.e., organophosphates, carbamates, organochlorines, synthetic pyrethroids); (4) plasticizers and chemicals produced by incomplete combustion of polyvinyl chloride (PVC), paper and putrescible substances (i.e., dioxin); (5) industrial substances and their by-products (i.e., phenols, dioxins, heavy metals, perfluorooctanoic acid, flames retardants) (30, 31).

The timing of EDC exposure should be considered when assessing their effects on the organism; especially in the initial stages of development, when the mammalian organism is extremely sensitive to disturbing agents, exposure might have more pronounced and long-lasting effects (3, 14, 16, 20, 22, 32). It is therefore unsurprising that prenatal exposure may result in significant changes. The effects of EDC on ovarian development have mainly been investigated using animal models or *in vitro* systems. Collectively, the collected data in the literature have shown that some EDC exposures damage the developing ovary by interfering with germ cell nest destruction, meiosis, follicle formation and vitality (11, 16, 22, 32). Early postnatal exposure to EDCs may results in significant changes in genetic transcription of somatic cells, altering the proper timing of physiological process of puberty which may be delayed or anticipated (4). Furthermore, EDCs appears to have a consistent role in decreasing both male and female fertility; testicular hypotrophy and ovarian polycystic syndrome (PCOS) seems to be associated to EDCs exposure (4, 11). In particular, EDCs seems to have a causative role in the onset of testicular dysgenesis syndrome (TDS) (1, 4, 22). Impaired spermatogenesis, testicular cancer, undescedent testis and hypospadias may be considered the symptoms of a developmental disorder, TDS, an increasing disease due to adverse environmental influences. Experimental and epidemiological studies show that TDS is the result of an interruption of physiological embryonal programming and gonadal development during fetal life (33, 34). Generally, in the most severe forms low serum testosterone is observed (35) and an increased risk of testicular cancer compared to milder forms has been highlighted (4, 36). Phthalates, vinclozolin, acetaminophen, and polybrominated diphenyl ethers (PBDE) seem to play a significant etiopathogenetic role in the onset of TDS (4, 37, 38). In female, there is little evidence regarding the effects of EDCs on fertility. Nevertheless, this data seems to suggest that long-term exposure to EDCs may alter female fertility (39). In particular, EDCs appears to have an etiopathogenetic role in the onset of endometriosis and of ovarian pathology (40). Phthalates, diethylstilbestrol, bisphenol A (BPA), TCCD may be involved in the onset of endometriosis occurring in 10% of fertile women, causing infertility in 50% of affected subjects (5, 40). Ovarian pathology seems to be related to the exposure to: PCB, phtalates, atrazine, genistein, BPA, TCDD, parabens, triclosan, dichlorodiphenyltrichloroethane (DDT), and metoxychloride (MXC) (39, 41). Even in males, reproductive function may affected by pollutants and EDCs, but evidence is scarce; EDCs, such as phthalates, bisphenol A, biphenyls, and vinclozolin, widespread use of therapeutic drugs, obesity and sedentary life-style may play a crucial role in this supposed decrease of male fertility (3–5, 38, 42).

## Adrenal Gland

Only a few studies have assessed the effects of EDC on the adrenal gland, especially concerning the risks related to exposure to chemicals. This paucity of evidence is not easily comprehensible, considering that the proper functionality of HPA axis is necessary for human life and it is a common target for many drugs and chemicals. Indeed, adrenal glands present some structural and biochemical features that make them ideal targets for EDCs, such as an elevated blood flow, lipophilic structure due to the high content in polyunsaturated fatty acids in cell membrane, and presence of CYP 450 enzymes producing toxic metabolites and free radicals (2, 43).

The most studied effect of EDCs on this axis is represented by their ability to interfere with the biosynthesis and metabolism of steroidal hormones through the modulation or the inhibition of enzymes involved in steroidogenesis. Aromatase, 5-α reductase, as well as 3-β, 11-β, 17-β hydroxysteroid dehydrogenases have a crucial role in metabolic pathways of adrenal steroidogenesis and their proper function is affected by EDCs, in particular xenoestrogens impair adrenal function inhibiting these enzymes (7, 17). Also, Steroid Acute Regulatory Protein (StAR), which regulates the first step of adrenal steroidogenesis, is a target of EDCs (44). Hundreds of chemicals and drugs may interact with HPA axis and every step of steroidogenesis may be affected by EDCs as well as each chemical disruptor may act altering different step of steroidogenesis (45). In evaluating the pathologic effects of EDCs on HPA axis, it should be considered that even a partial impairment of proper adrenal function may have severe effects on human health. In this perspective, bioaccumulation of these chemicals in adipose tissue might generate a “cocktail,” with clinical effects that may be observed only after several years of constant, low-dose exposure (17). Hexachlorobenzene is among the chemicals which is able to disrupt corticoid hormone function in Wistar rats (5, 43).

## Thyroid Gland

Several environmental chemical substances are known to alter iodine absorption by inhibiting the Sodium-Iodide symporter channel (NIS): in particular, perchlorate and thiocyanate are able to affect thyroidal metabolism inhibiting NIS (7). This channel transports iodine inside thyrocytes and, considering the pivotal role played by iodine in the biosynthesis of thyroid hormones, it is comprehensible that an alteration of NIS may impair thyroid function (2). High levels of perchlorate are present in explosives, fertilizers, and in air-bags (7). A survey performed by National Health and Nutrition Examination Survey (NHAMES) 2001–2008 suggests its presence also in foods, such as milk, vegetables, fruits, eggs in USA (2). Thiocyanate is present in cigarette smoke and in Brassicaceae. Braverman et al. observed 3,100 subjects exposed to perchlorate, thiocyanate, and nitrates; this exposure resulted in a significative decrease of free thyroxine levels, which was more marked in pubertal subjects and it was not associated to increase of TSH levels. Perchlorate, thiocyanate, and nitrates are widespread and their presence is not limited to industrial products, but many foods and potable water are contaminated by these EDCs. They are able to cause a loss of function of NIS binding to NIS and blocking iodide transporter. As a consequence, a lower iodine bioavailability may results in hypothyroidism, even more if the exposure to high doses is prolonged in certain area affected by iodine deficiency. No conclusive result can be drawn from current data, but iodine supplementation in pregnancy and in children may protect from the action of these EDCs. Further studies should investigate the dose-effects relationship in these regards (46).

## EDCs and Endocrine Glands Cancer

Several scientific institutions (European Commission, European Environmental Agency, The Endocrine Society, WHO/UNEP, IARC) studied the association between EDCs and cancer affecting testis, prostate, thyroid and breast suggesting that the exposure to some EDCs may be a risk factor in the development of these tumors (2, 47–49). Fungicides, pesticides, PDBE, organochlorides, PCB, DDT, dichlorodiphenyldichloroethylene (DDE), arsenic, and cadmium have been proposed to play a role in testicular cancer with etiopathogenetic role in the onset of TDS. Otherwise, pesticides, TCDD, PCB, and solvents appears to be involved in thyroid cancer. Several other compounds are able to impair thyroid function; among the many EDCs, biocides are worth mentioning. “Biocide” is the definition applied to any substance or mixture in the form in which it is supplied to the user, consisting of, containing or capable of generating one or more active substances, for the purpose of destroying, eliminating and rendering harmless, preventing action or exercising other control effect on any harmful organism, by any means other than mere physical or mechanical action. Zeng et al. used data from a Connecticut case-control study and examined the association with occupational exposure to biocides and pesticides. Detailed information was collected on the name of the job, the tasks performed, the name of the company and the type of industry, starting and ending dates of the subjects during their life for at least 1 year. There was a significantly increased of thyroid cancer risk associated with exposure to pesticides and biocides (OR = 1.65; IC 95% 1.16–2.35) and this risk was more marked in male adults (OR = 3.11; IC 95% 1.25–7.72) (50). PCB, dioxins, cadmium, phytoestrogens, DES, furans, ethylene oxide may contribute in the development of breast cancer. Finally arsenic, cadmium, PCB, and pesticides seem to contribute importantly to prostate carcinogenesis (5). A recent study led by Benedetti et al. investigated the incidence of these tumors in certain polluted areas in Italy (the Italian National Priority Contaminated Sites (NPCSs). A significantly higher incidence of breast and prostate cancers was observed in these areas; nevertheless, incidence of breast cancer seems to be increased in each area, whereas incidence of prostate cancer appears to increase in certain areas and to decrease in others (51). This data suggests that a wide range of chemicals may contribute to development of breast cancer whereas specific chemical may affect prostate carcinogenesis. Otherwise, the scarce number of cases of testicular and thyroid cancer make impossible to draw conclusions regarding the role of EDCs in these pathologies.

## A Model of Endocrine Disruption: AhR

As aforementioned, EDCs may bind to and activate or block several endocrine receptors (5, 52), acting as total, partial or inverted agonist or antagonist. The ability to bind AhR with its consequent effects represents a well-known action of EDCs; AhR is a cell nuclear receptor and acts as ligand-activated transcription factor that senses the presence of foreign compounds such as Persistent Organic Pollutants (POPs) and leads to the activation of cytochrome P450 enzymes needed for their clearance from the body, being involved in modulating the response to xenobiotics (xenobiotic responsive elements, XREs) (53, 54). Besides the detoxification and bioactivation of XREs, AhR has been shown to regulate expression of enzymes necessary to the metabolism of endogenous substances (i.e., hormones) and to modulate a much wider range of cellular pathways involving proliferation, differentiation, apoptosis (5). Tetrachlorodibenzo-P-dioxin (TCDD), a well-known AhR ligand, has remarkable carcinogenic activity, suggesting that AhR activation may play a role in carcinogenesis (5, 14, 52). Furthermore, AhR may be activated by several EDCs, such as PCBs, dioxins, furans, and polycyclic aromatic hydrocarbons, albeit with different potency (18, 53, 55). In absence of its ligands, AhR lies in cytoplasm assisted by a complex of co-chaperone proteins (54, 56). In particular, AhR is assisted in cytoplasm by co-chaperone AhR-interacting protein (AIP). AIP plays a pivotal role in regulating the response to XREs; as a matter of fact, its mutation brings about the loss of its function and consequently an alteration of biological pathways controlled by AhR causing an impairment in the response to XREs (53). Otherwise, in the presence of its ligands, AhR enters the nucleus and binds to its nuclear translocator, known as AhR nuclear translocator (ARNT), and to its coactivators creating a molecular complex whose function is to bind XREs activating specific target-genes (38, 54, 57). Besides the detoxification and bioactivation of XREs, AhR has been shown to regulate expression of enzymes necessary to the metabolism of endogenous substances (i.e., hormones) and to modulate a much wider range of cellular pathways involving proliferation, differentiation, apoptosis (5). The loss of function of AIP caused by its mutation showed a marked association with Familiar Isolated Pituitary Adenomas (FIPAs); in particular it has been observed that FIPAs show unfavorable response to medical therapies, greater severity and earlier age of onset (58, 59). Some evidence showed the association between EDCs and the development of FIPA, highlighting that AhR may be targeted by EDCs with mutagenic properties (55); in particular, the association between a significant exposure to dioxins, able to bind AhR, and an increased incidence of FIPA has been investigated (60). Pesatori et al. showed that the massive exposure to dioxins caused by industrial pollution due to 2,4,5 trichlorophenol explosion in a chemical factory occurred at Seveso in 1976 was not accompanied by a significative increased incidence of FIPA (14). Nevertheless, Authors reported an increased number of cases of pituitary adenomas in exposed subjects, including not familiar pituitary adenomas and prolattinoma (61). Cannavò et al. focused their attention on the prevalence of GH-secreting adenomas observing an higher prevalence of acromegaly in the most polluted areas which could not be explained by familiar or genetic causes, and suggesting that chemicals may play a role in the pathogenesis of pituitary adenoma (62, 63).

## Conclusion

In scientific literature the hazardous effects of environmental EDCs on endocrine system and their complex mechanisms of regulation have been reported with supporting evidence. However, further long-term studies performed on wide number of subjects are necessary to verify and assess the hypothesized causal relationship between EDCs and endocrine pathology. The aim should be represented by the detection of the substance, of its mechanism of action, the explanation of relationship dose-effect clarifying the causal link between the substance and pathology. Phenomena such as bio-accumulation and trans-generational inheritance are clear obstacles to research and new strategies in these regards should be pursued.

## Author Contributions

All authors listed have made a substantial, direct and intellectual contribution to the work, and approved it for publication.

### Conflict of Interest Statement

The authors declare that the research was conducted in the absence of any commercial or financial relationships that could be construed as a potential conflict of interest.
